# The small subunit of DNA polymerase D (DP1) associates with GINS–GAN complex of the thermophilic archaea in *Thermococcus* sp. 4557

**DOI:** 10.1002/mbo3.848

**Published:** 2019-05-08

**Authors:** Shuhong Lu, Xuesong Zhang, Kaiying Chen, Zimeng Chen, Yixiang Li, Zhongquan Qi, Yulong Shen, Zhuo Li

**Affiliations:** ^1^ College of Ocean and Earth Sciences Xiamen University Xiamen Fujian China; ^2^ Third Institute of Oceanography, Ministry of Natural Resources of China Xiamen Fujian China; ^3^ School of Medicine Guangxi University Nanning Guangxi China; ^4^ State Key Laboratory of Microbial Technology Shandong University Qingdao Shandong China

**Keywords:** archaea, DNA replication, GAN, GINS, PolD

## Abstract

The eukaryotic GINS, Cdc45, and minichromosome maintenance proteins form an essential complex that moves with the DNA replication fork. The GINS protein complex has also been reported to associate with DNA polymerase. In archaea, the third domain of life, DNA polymerase D (PolD) is essential for DNA replication, and the genes encoding PolDs exist only in the genomes of archaea. The archaeal GAN (GINS‐associated nuclease) is believed to be a homolog of the eukaryotic Cdc45. In this study, we found that the *Thermococcus* sp. 4557 DP1 (small subunit of PolD) interacted with GINS15 in vitro, and the 3′–5′ exonuclease activity of DP1 was inhibited by GINS15. We also demonstrated that the GAN, GINS15, and DP1 proteins interact to form a complex adapting a GAN–GINS15–DP1 order. The results of this study imply that the complex constitutes a core of the DNA replisome in archaea.

## 1 INTRODUCTION

DNA replication is an essential biological process in all domains of life (Ishino & Ishino, 2012; Masai, Matsumoto, You, Yoshizawa‐Sugata, & Oda, 2010; Mott & Berger, 2007; Nagata et al., 2017), and replicative DNA polymerases play key roles in the DNA replication process to ensure accurate replication and transmission of genetic information. In Euryarchaea, the main DNA polymerases belong to the polymerase B (PolB) and D (PolD) families (Makarova, Krupovic, & Koonin, 2014). Members of the PolD family are heterodimers consisting of one small subunit, DP1, and one large subunits, DP2. DP1 is responsible for 3′–5′ proofreading activity while DP2 possesses 5′–3′ polymerization activity (Raia et al., 2019; Takashima et al., 2019). GINS is a central protein complex involved in the initiation of DNA replication that is believed to move along with the replication fork. The eukaryotic GINS complex is composed of Sld5, Psf1 (partner of Sld5‐1), Psf2, and Psf3 (partner of Psf2‐3) (Takayama et al., 2003). The CMG (Cdc45–MCM–GINS) complex moves along the DNA replication fork and is essential to the growth of eukaryotic cells (Gambus et al., 2006; Moyer, Lewis, & Botchan, 2006; Pacek, Tutter, Kubota, Takisawa, & Walter, 2006). In the archaea genome, two genes are known to encode GINS15 (homolog of Sld5 or Psf1) and GINS23 (homolog of Psf2 or Psf3) (Marinsek et al., 2006). Based on a primary sequence similarity search, the eukaryotic Cdc45 coding gene is absent from the archaea genome (Krastanova et al., 2012; Makarova, Koonin, & Kelman, 2012). GAN (GINS‐associated nuclease) has a secondary structure similar to that of Cdc45 and contains a 5′–3′ single‐stranded DNA (ssDNA)‐specific exonuclease domain, similar to bacterial RecJ nuclease. Association with GINS stimulates the nuclease activity of GAN (Li et al., 2011). It has been speculated that GAN is an archaeal version of eukaryote Cdc45 (Li et al., 2011; Nagata et al., 2017).

In Eukaryotes, GINS physically and functionally associates with DNA polymerase on the replication fork (Chang, Wang, Bermudez, Hurwitz, & Chen, 2007). Dpb2p, the non‐catalytic subunit of DNA polymerase epsilon (Pol ε), does not contain DNA extension and 3′–5′ nuclease activities, but does interact with Psf1 and Psf3 subunits of the GINS complex (Garbacz et al., 2015; Grabowska et al., 2014; Muramatsu, Hirai, Tak, Kamimura, & Araki, 2010; Sengupta, van Deursen, de Piccoli, & Labib, 2013; Takayama et al., 2003).

However, in archaea, the detailed mechanism and the cooperation within the GAN, GINS, and PolD complexes including the association patterns have yet to be identified. In this study, the relationships among the above DNA replication proteins in *Thermococcus* sp. 4557 were analyzed. We found that the DP1–GINS–GAN (PGG) complex constitutes the archaeal DNA replisome core. The data obtained in this study will improve our overall understanding of the mechanisms involved in archaeal DNA replication.

## 2 MATERIALS AND METHODS

### 2.1 Gene cloning and plasmid construction

Genes encoding PolD‐DP1 (AEK73338.1), DP2 (AEK73341.1), GAN (AEK72413.1), GINS15 (AEK72061.1), and GINS23 (AEK72908.1) were amplified by PCR using *Thermococcus* sp. 4557 genomic DNA as a template and the upstream and downstream primer. The oligonucleotides for cloning the above genes are listed in Appendix Table A1. The amplified fragments of all genes were digested with restriction enzymes and inserted into a modified pET15b vector [described in Shen et al. (2001)] to create recombinant pET15b/His_6_‐tagged gene fragments. The nucleotide sequences of the inserted DP1, DP2, GAN, GINS15, and GINS23 genes were confirmed by sequencing (Invitrogen, Shanghai, China). The plasmids expressing non‐tagged GAN and GINS15 proteins were generated using the single point mutation method provided by Cvent Scientific, China.

### 2.2 Expression and purification of recombinant proteins

Recombinant His_6_‐tagged DP1, DP2, GAN, GINS15, and GINS23 were produced in *Escherichia coli* strain BL21 (DE3)‐Codon Plus‐RIL grown in 1,000 ml Luria–Bertani (LB) medium containing ampicillin (100 µg/ml) and chloramphenicol (34 µg/ml). Cells were grown at 37°C until the OD600 reached 0.4. The expression was then induced by incubation in the presence of isopropyl β‐D‐1‐thiogalactopyranoside (1 mM) for 16 hr at 16°C. Cells were subsequently harvested and disrupted by sonication in buffer A (50 mM Tris‐HCl, pH 8.0, and 200 mM NaCl). Next, samples were incubated at 80°C for 20 min, and then centrifuged at 10,000×*g* for 10 min; after which, the soluble heat‐resistant fractions were precipitated with 80% saturated ammonium sulfate. The precipitated proteins were resuspended in buffer A and dialyzed against buffer B (50 mM Tris‐HCl, pH 8.0, and 100 mM NaCl) to remove the ammonium sulfate. After dialysis, the samples were loaded onto a 5‐ml HiTrapQ column that had been pre‐equilibrated with buffer B. The fractions containing the corresponding proteins were pooled and loaded onto a 5‐ml nickel nitrilotriacetic acid–agarose column that was subsequently washed with 10 column volumes of buffer A containing 40 mM imidazole and eluted with three column volumes of elution buffer containing 250 mM imidazole. The eluted fractions were pooled, concentrated, and then separated on a gel filtration column (Sephacryl S‐200).

### 2.3 Gel filtration assay

Aliquots of each experimental protein (100 µg) or protein mixture and gel filtration standards (BioRad) were diluted in 200 µl of 25 mM Tris‐HCl (pH 7.5), 100 mM NaCl, and 10% (v/v) glycerol and loaded onto a Superdex‐200 column (HR10/30; GE Healthcare) that had been pre‐equilibrated in the same buffer. Fractions (250 µl) were collected from the column at a flow rate of 0.5 ml/min. The proteins present in aliquots (80 µl) of each fraction were separated by electrophoresis through a 12% (w/v) polyacrylamide–SDS gel and stained with Coomassie brilliant blue (R250).

### 2.4 Exonuclease assay

The DNA substrate 5′‐CGAACTGCCTGGAATCCTGACGACATGTAGCGAACGATCACCTGA‐3′ with Cy5 labeled at the 5′ end was used to test the exonuclease activity of DP1. The reaction was conducted in a mixture (20 µl) containing 0.5 pmol labeled DNA substrates, 25 mM Tris‐HCl (pH 8.0), 125 µg/ml bovine serum albumin, 1 mM dithiothreitol (DTT), 1 mM MnCl_2_, and proteins as indicated. Reactions were conducted at 60°C for 1 hr, after which they were stopped by the addition of an equal amount of the stop buffer (95% formamide, 10 mM EDTA and 0.1× Tris‐Borate‐EDTA). The samples were then boiled for 2 min, immediately chilled on ice, and electrophoresed on a 15% polyacrylamide gel containing 8 M urea. The gels were visualized by scanning using the Cy5 channel using a Typhoon 9410 scanner (Amersham).

### 2.5 Pull‐down assay

His_6_‐tagged DP1 (100 µg) and an equal amount of untagged GINS15, untagged GAN, or untagged GINS15, and untagged GAN were mixed in a buffer containing 50 mM Tris‐HCl (pH 8.0) and 500 mM NaCl on ice for 30 min. The mixture was then combined with 20 µl of nickel nitrilotriacetic acid–agarose slurry that had been pre‐equilibrated in 50 mM Tris‐HCl (pH 8.0) and 500 mM NaCl. The agarose beads were collected by centrifugation at 500×*g* for 1 min, after which they were washed five times at room temperature in 500 µl wash buffer (50 mM Tris‐HCl, pH 8.0, 500 mM NaCl, 50 mM imidazole). Next, 50 µl of SDS–PAGE loading buffer (250 mM Tris‐HCl, 0.5% SDS, 50% glycerol, 0.5% bromophenol blue, and 0.5 mM DTT) was added to the samples, which were then boiled at 100°C for 15 min and separated on a 15% SDS–polyacrylamide gel that was subsequently stained with Coomassie bright blue. His_6_‐tagged DP1, untagged GINS15, untagged GAN, or untagged GINS15, and GAN mixture were also loaded separately as controls.

### 2.6 ELISA detection

DP1, GINS, or GAN (1 µg/100 µl of each protein) was fixed onto the surface of the wells shown in Figure 5 (left panel, red), and incubated at room temperature for 2 hr. Skim milk (5%, with PBS, 100 µl/well) was then applied, after which the wells were incubated at room temperature for 2 hr. The binding proteins (100 µl) shown in Figure 4 (left panel, black) were added into wells, after which samples were incubated and washed as described above. His‐HRP antibody (1:1,000 diluted) was then added into wells, the samples were incubated at room temperature for 2 hr, and then washed with phosphate buffered saline with tween 20 buffer five times to remove unbound proteins. TMB (3, 3′, 5, 5′‐tetramethylbenzidine) (TMB Single‐Component Substrate Solution from Solarbio) was added into wells (100 µl/each well) and incubated at 37°C for 10 min, the reaction was stopped by adding 50 µl 0.5 N HCl, and the absorbance at 460 nm was measured.

### 2.7 Surface plasmon resonance analysis

The interaction between GINS23, GINS15, and DP1 was confirmed using a BIAcore 3000 system (GE Healthcare, Hatfield, UK). This method is based on surface plasmon resonance (SPR). All experiments were conducted at 25°C and a flow rate of 30 µl/min. Recombinant GINS23 was dissolved in sodium acetate buffer (pH 4.5) and amine coupled onto a CM5 sensor chip (GE Healthcare) following the manufacturer's instructions. Phosphate‐buffered saline with 0.005% Tween 20 was used as a running buffer. First, GINS15 (50 nM) was injected onto GINS23. After the signal stabilized at about 380 s, the DP1 (50 nM) was injected onto the sensor chip to observe the binding. As a control, DP1 (50 nM) was injected directly onto GINS23 at 580 s in another test. After each measurement, the surface was regenerated with 10 mM glycine hydrochloride (pH 2.5).

## 3 RESULTS

### 3.1 Cloning, expression, and purification of proteins

**Figure 1:**
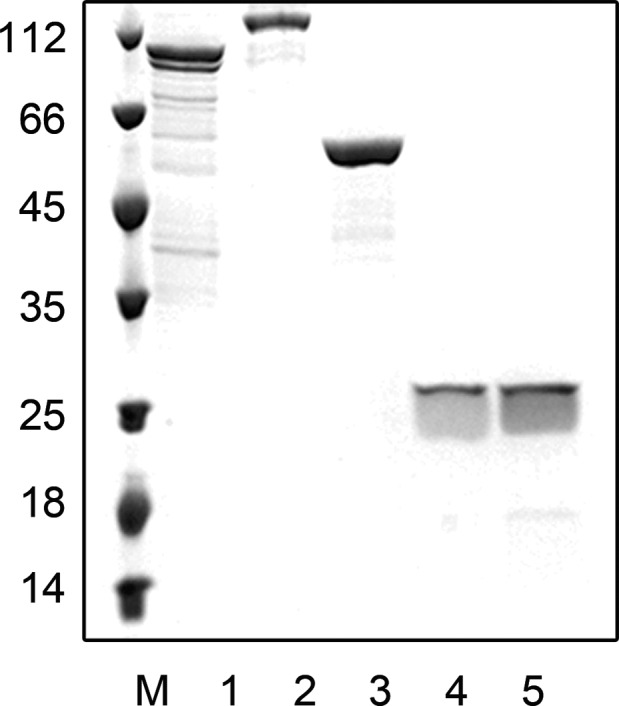
SDS–PAGE analysis of purified recombination proteins. Lane 1, Marker; Lane 2, PolD‐DP1 (79 kDa); Lane 3, PolD‐DP2 (147 kDa); Lane 4, GAN (51.2 kDa); Lane 5, GINS15 (22.8 kDa); Lane 6, GINS23 (19.2 kDa)

To understand the relationship between PolDs, GINS, and GAN proteins, and their biochemical characters, we cloned the individual genes encoding the above proteins from *Thermococcus* sp. 4557, a strain isolated from the hydrothermal vent area of the Gulf of California (Wang, Gao, Xu, & Ruan, 2011). Each gene was expressed in *E. coli* cells, and the recombinant proteins were purified as described in the Materials and Methods. The results of purification, as analyzed by SDS–PAGE, are shown in Figure 1. The molecular sizes are in agreement with calculated molecular weights.

### 3.2 DP1–DP2 and GAN–GINS15 form stable complexes

**Figure 2:**
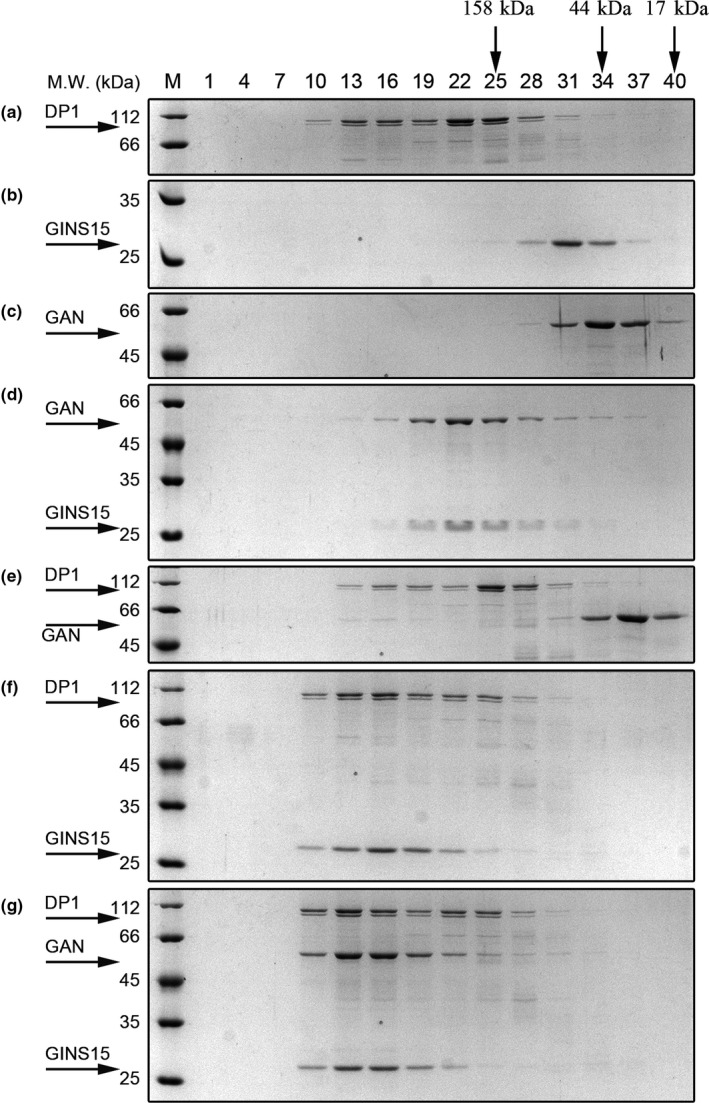
Analysis of the interaction among PolD proteins, GAN, and GINS proteins by gel filtration. A sample containing 100 µg of each protein (indicated on the right of the corresponding panels, A through G) was subjected to Superdex‐200 gel filtration analysis. Aliquots (10 µl) of each fraction were separated by electrophoresis with a 15% (w/v) polyacrylamide–SDS gel. The gel was stained with Coomassie brilliant blue. More detailed SDS–PAGE images are available in Appendix Figure A1

**Figure A1:**
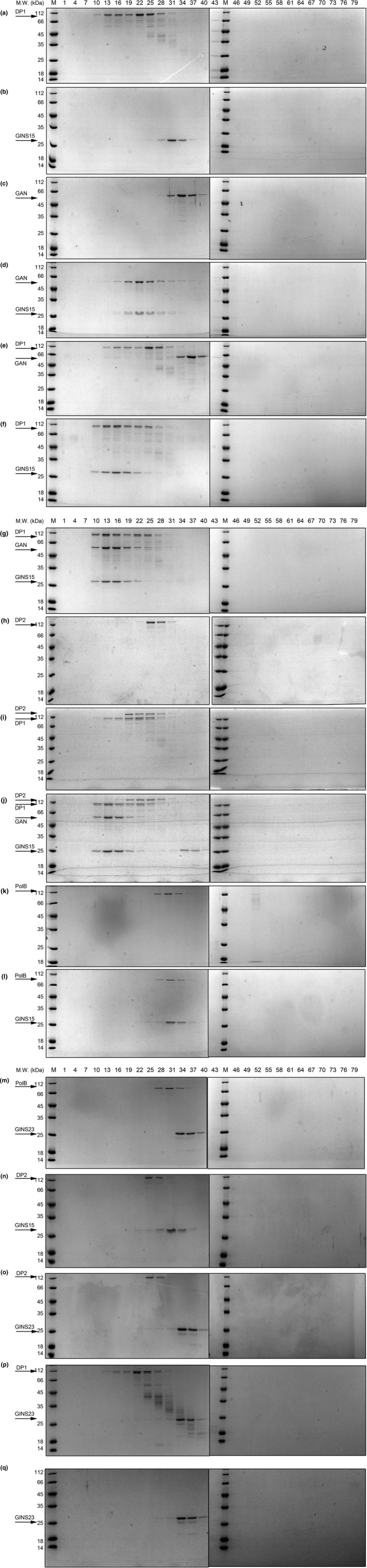
Size exclusion assay followed by SDS–PAGE of DNA replication proteins

To investigate the PolD‐associated replication complex, we tested the individual interactions between DP1, DP2, GINS15, GINS23, and GAN using the size exclusion column followed by SDS–PAGE assay. As shown in Figure 2, DP1, DP2, GAN, GINS15, and GINS23 were eluted in different peak fractions when there was a single protein in the test sample, and the observed sizes of these proteins were in accordance with their molecular weight. As expected, DP1 eluted as both dimer and monomer while DP2 eluted as monomer. DP1 and DP2 (Appendix Figure A1) directly eluted together in the same peak fraction, and co‐elution of GINS15 and GAN (Figure 2d) was also observed in agreement with the results of previous studies (Li et al., 2011; Shen, Tang, Matsui, & Matsui, 2004; Shen, Tang, & Matsui, 2003; Takashima et al., 2019). One exception is GAN from *Thermococcus* sp. 4557 eluted at the peak of fraction 34, corresponding to monomer, different from our previous study that GAN from *Thermococcus kodakarensis* exist as both dimer and monomer (Li et al., 2011). The results also showed that the interactions among the above proteins were conserved within the species of Thermococcales.

### 3.3 GINS15 interacts with DP1

In our previous study, PolD was reported to potentially exist in the same complex containing GINS and GAN subunits in *T. kodakarensis* (Li, Santangelo, Cubonova, Reeve, & Kelman, 2010). Also using the pull‐down technique, subsequent research further reported the co‐purification of GINS15 and DP1 in *Pyrococcus abyssi* (Pluchon et al., 2013). Since proteins directly interacting with and regulating the activity of PolD have not been studied extensively, it is not clear whether PolD proteins associate with GINS, and if so, which subunit of PolD directly mediates the association with the GINS complex. To answer these questions, we tested the interactions between DP1, DP2, GINS15, GINS23, and GAN in a series of possible combinations by a gel filtration assay. As shown in Figure 2a–c, DP1, GINS15, and GAN were eluted in the peaks of fractions 13 and 22, 31, and 34, respectively. Interestingly, DP1 and GINS15 were coeluted in fraction 16 (Figure 2f). When compared with the individual elution peaks of DP1 and GINS15, which were in fractions 22 and 31, respectively, it is plausible to conclude that DP1 and GINS15 directly interact with each other. However, incubation of DP1 and GAN did not result in formation of a complex (Figure 2e). When DP1, GAN, and GINS15 were mixed and analyzed in the same assay, a complex was eluted in fraction 13 (Figure 2g), which was earlier than that of DP1–GINS15 (fraction 16). These findings indicate that DP1 interacts directly with GINS15, which also directly interacts with GAN. In other words, GINS15 acts as a scaffold binding both DP1 and GAN to form a DP1‐GINS15‐GAN complex.

### 3.4 Organization of the DP1–GINS–GAN complex in vitro analyzed by pull‐down assay

**Figure 3:**
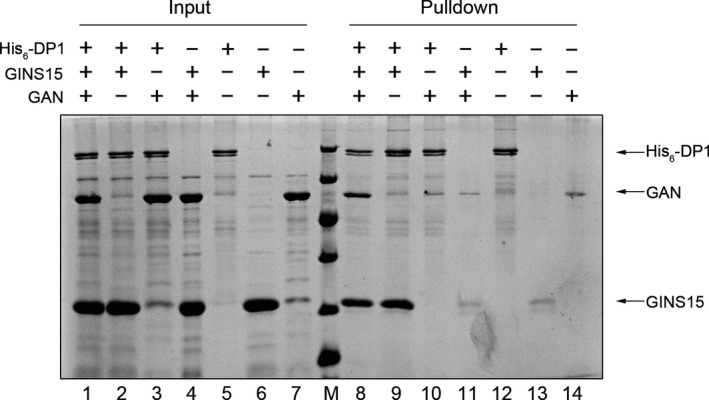
Analysis of physical interaction among DP1, GINS, and GAN in a pull‐down assay. Lanes 1–7: input samples; lane 8: protein size marker; lanes 9–14: elution fraction

Since the results from size exclusion showed that GINS15 interacts with DP1 and GAN at the same time, it is reasonable to speculate that the complex adopts a DP1–GINS15–GAN order. To further confirm this, we conducted a His_6_‐tagged DP1‐mediated pull‐down assay in vitro. Various protein combinations as inputs were analyzed by SDS–PAGE (lanes 1–7, Figure 3). After incubation, unbound proteins were washed out and the proteins eluted from the Ni‐column were analyzed by SDS–PAGE (lanes 8–14, Figure 3). As shown in lane 9 (Figure 3), the His_6_‐tagged DP1 pulled down the untagged GINS15. In the mixture containing His_6_‐tagged DP1, untagged GINS15, and GAN (lane 8), the three proteins were eluted together. Additionally, His_6_‐tagged DP1 was unable to pull‐down GAN directly, suggesting that DP1 does not directly associate with GAN (lane 10). For the control GINS and GAN, single His_6_‐DP1, single GINS, and single GAN (lanes 11–14, respectively), although weak GAN or GINS bands were observed at the corresponding positions (lanes 11, 13, and 14), the band intensities were far weaker than those in lanes 8 or 9. The pull‐down assay confirmed that the three proteins formed a complex in the order of DP1–GINS15–GAN.

### 3.5 Confirmation of the interaction among DP1, GINS, and GAN using the ELISA assay

**Figure 4:**
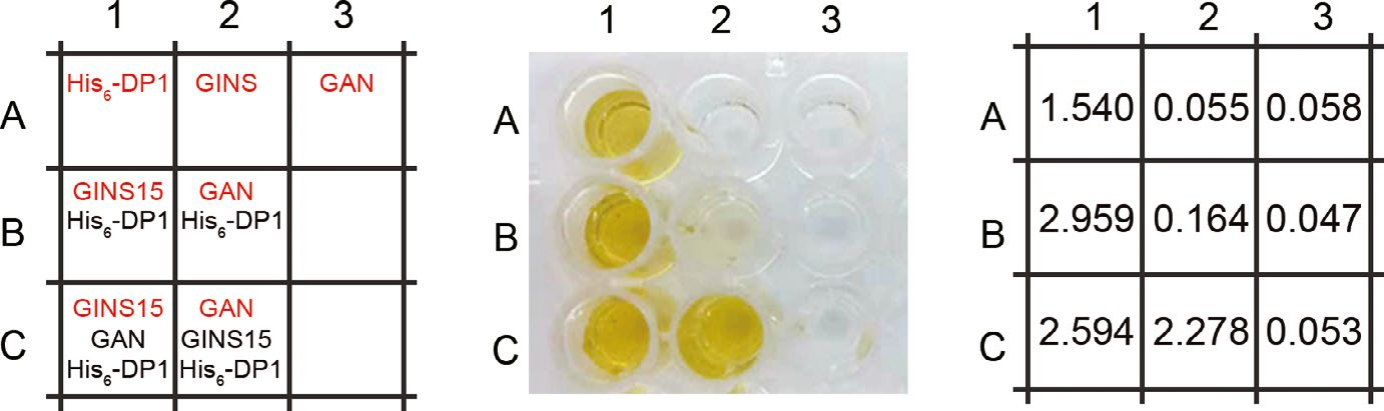
Analysis of protein–protein interactions by ELISA. Wells A1, A2, and A3 in all panels are the controls. The left panel showed sample components in each well of the plate. Proteins in red were added into wells first as the coating proteins, while those in black were subsequently added into each well to detect association with the corresponding coated protein. The middle panel shows coloration of the ELISA plate. The right panel indicates the absorbance at 460 nm corresponding to each well. Measurements and calculations are available in Appendix Figure A2

To further verify the relationship among DP1, GINS15, and GAN, an ELISA assay was performed. As shown in Figure 4, proteins (red) were fixed on the surface in each well. The samples were then incubated (Figure 4, left panel), after which unbound proteins were washed out. The samples in all wells were developed by His‐HRP antibody and followed by measuring absorbance at 460 nm (A460). The presence and absence of His_6_‐tagged DP1 are indicated by yellow and transparent wells, respectively (Figure 4, middle panel). The yellow color in B1 supports that GINS15 associates with His_6_‐DP1. However, when His_6_‐DP1 was added into well B2 with fixed GAN, no yellow color was observed, indicating that there is no direct interaction between GAN and DP1. In C2, which contained GAN, DP1, and GINS15, a yellow color appeared. Moreover, measurement at the A460 (Figure 4, right panel) was in agreement with the coloration results. Taken together, these findings again support that GINS15 serves as a linker between DP1 and GAN to form a DP1–GINS15–GAN complex.

### 3.6 GINS23–GINS15–DP1 forms a stable complex

**Figure 5:**
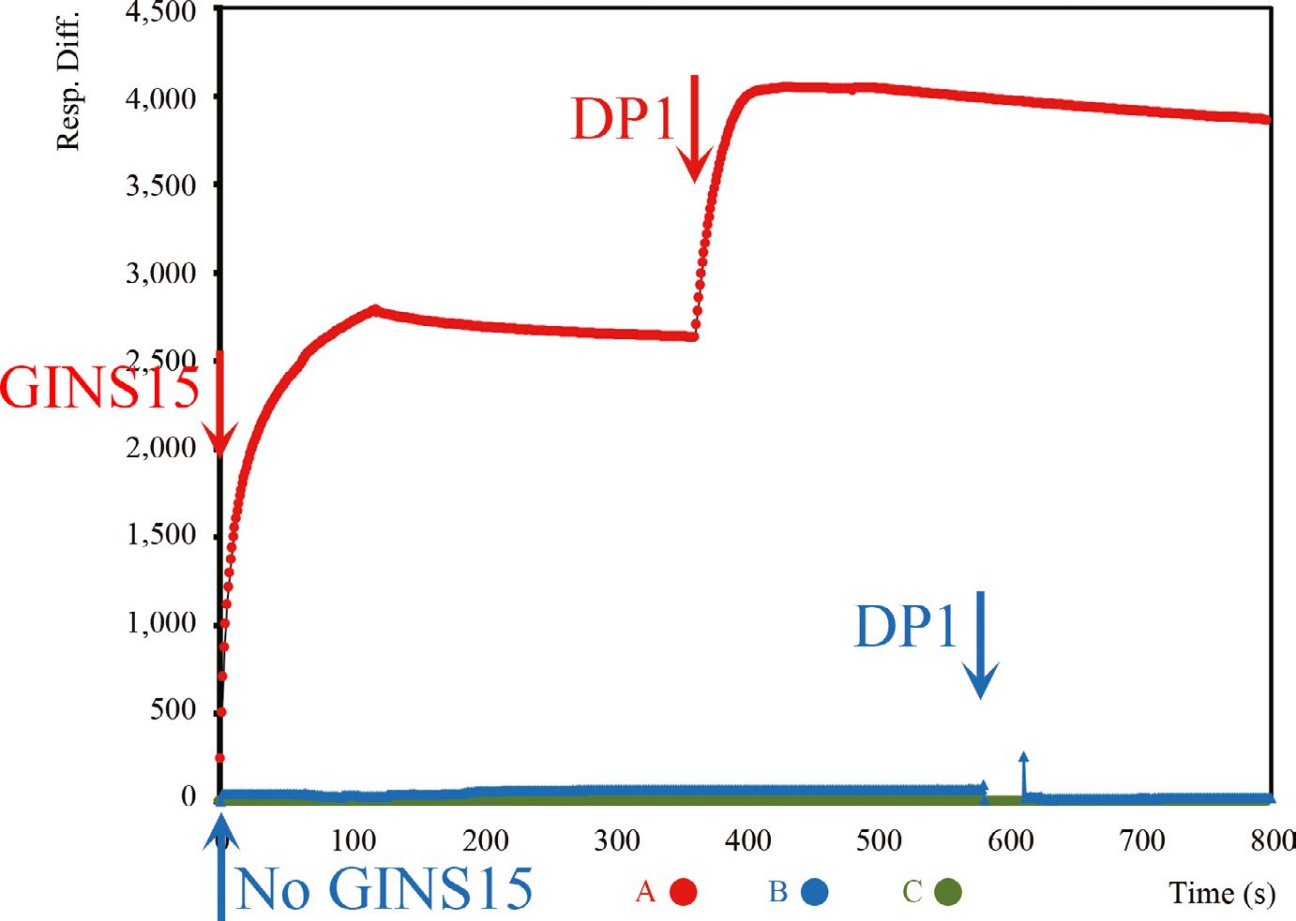
Analysis of the interaction by surface plasmon resonance (SPR) assay. Recombinant proteins were used in this assay. GINS23 was immobilized on a CM5 BiaCore sensor chip. The red curve shows that GINS15 was injected at 0 s and DP1 was injected at 380 s, while the blue curve shows that CM5 running buffer was injected at 0 s and DP1 was injected at 580 s. The green curve indicates the background

The archaeal GINS complex is composed of two copies of GINS15 and GINS23 (MacNeill, 2010); therefore, it is necessary to investigate whether GINS23 forms a complex with GINS15‐DP1 as well. To further test the association order between DP1 and GINS15‐23 proteins, we conducted a SPR assay (Figure 5). First, GINS23 was bound to the CM5 Chip, after which GINS15 was added into the buffer at 0 s. A corresponding peak shows the interaction between GINS15 and GINS23, which is in agreement with the results of a previous report. When DP1 flowed through at 380 s following the addition of GINS15, a higher peak was observed (red curve). These findings clearly demonstrate that GINS23, GINS15, and DP1 constitute a tight complex. When GINS15 was omitted, no peak appeared with the addition of DP1 at 580 s to bound GINS23 (blue curve), indicating that there is no direct interaction between DP1 and GINS23. The above observation supports the interaction between GINS15 and DP1 is not interrupted by the association between GINS15 and GINS23. In other words, the GINS complex interacts with DP1 complex through binding between GINS15 and DP1 in the order of GINS23‐GINS15‐DP1.

### 3.7 **GINS15 inhibits the DNA 3**′**–5**′** exonuclease activity of DP1**

**Figure 6:**
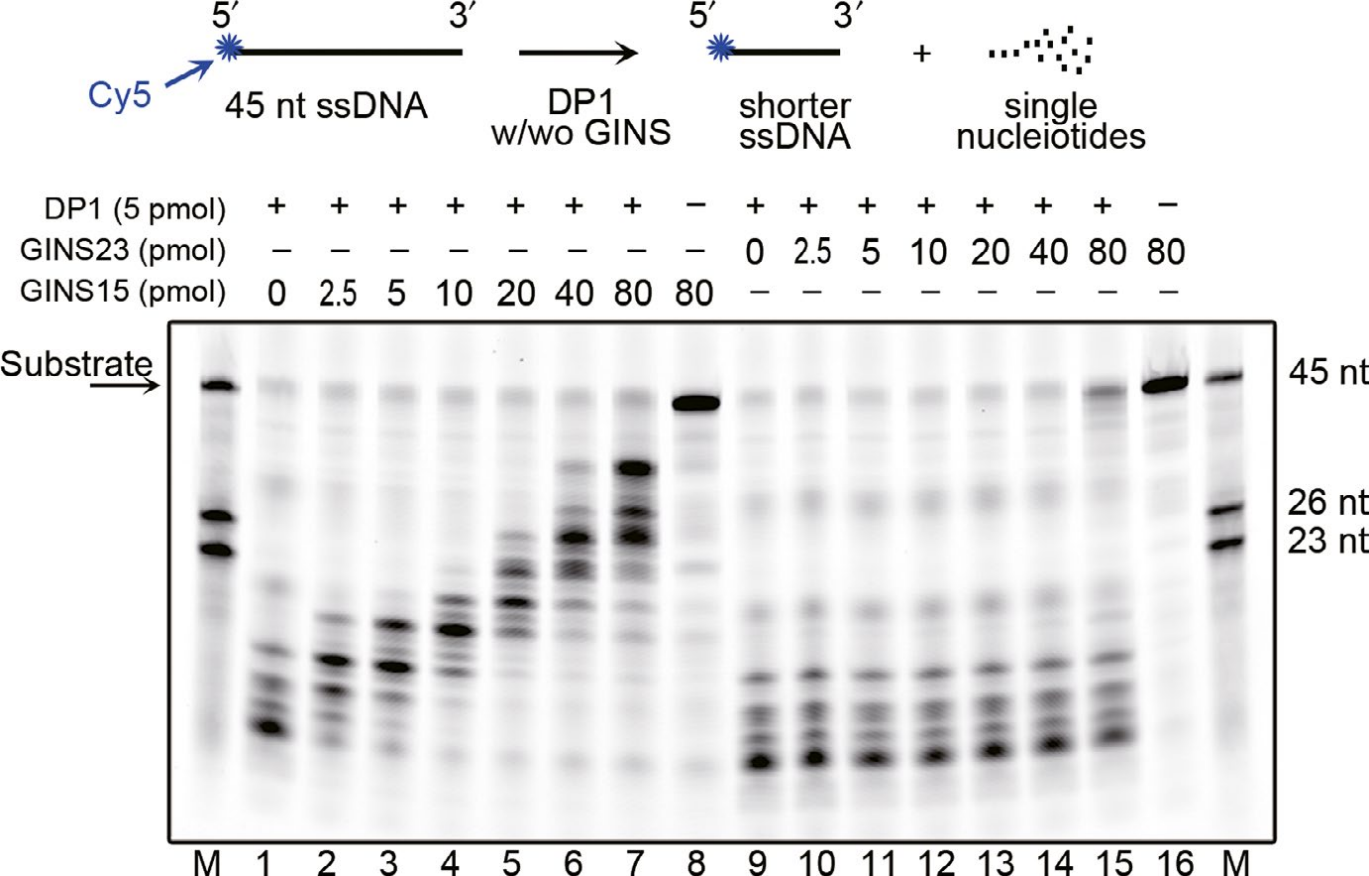
3′–5′ nuclease activity of the DP1 subunit of PolD was inhibited specifically by GINS15, but not GINS23. The upper panel illustrates the labeled substrate and reaction. Exonuclease assays were performed in 20 µl reactions containing 0.5 pmol substrates, 25 mM Tris‐HCl (pH 8.0), 1 mM MnCl_2_, 125 µg/ml BSA, and GINS15 or GINS23 proteins as indicated. Reactions were incubated at 60°C for 1 hr. Following incubation, reactions were stopped by adding 20 ml of 95% formamide, 10 mM EDTA, and 0.1× Tris‐Borate‐EDTA (TBE) followed by incubation at 95°C for 2 min

The DP1 subunit of PolD is a proofreading exonuclease responsible for the fidelity of DNA polymerase (Cann, Komori, Toh, Kanai, & Ishino, 1998; Ishino, Komori, Cann, & Koga, 1998; Uemori, Sato, Kato, Doi, & Ishino, 1997). Because we demonstrated that GINS15 interacts with DP1 as described above, it was necessary to determine if GINS is involved in the biochemical activities of DP1; therefore, we tested the effects of GINS15 and GINS23 on the DNA 3′–5′ exonuclease activity of DP1. As shown in Figure 6, a Cy5‐labeled single‐stranded deoxy‐oligonucleotide (45 nt) was used as a substrate to test the 3′–5′ exonuclease activity of DP1 (Figure 6, upper panel). As the concentration of GINS15 increased, the exonuclease activity of DP1 was suppressed (Figure 6, lanes 1–8). At the same time, GINS23 had neither stimulatory nor inhibitory effects on the exonuclease activity of DP1 (Figure 6, lanes 9–16). Different length DNA fragments (45, 26, and 23 nt) were used as markers (indicated on both sides of gel in Figure 6). The results indicated that GINS15, but not GINS23, physically and biochemically interacts with DP1.

## 4 DISCUSSION

Based on their amino acid sequence similarity, DNA polymerases can be classified into seven families (A, B, C, D, E, X, and Y) (Braithwaite & Ito, 1993; Cann & Ishino, 1999; Tori, Kimizu, Ishino, & Ishino, 2007). In eukaryotes, replication of the nuclear genome is largely conducted by three members of the B family of DNA polymerases, polymerases α, ε, and δ (Pols α, ε, and δ, respectively). Polymerase α mainly synthesizes short RNA–DNA primers to initiate replication, while Pols ε and δ are responsible for DNA chain elongation (Bell & Labib, 2016; Burgers & Kunkel, 2017; M. A. Garbacz et al., 2018; Lujan, Williams, & Kunkel, 2016; Stodola & Burgers, 2017). One model supports that Pol ε replicates the leading DNA strand, while the lagging strand is primarily conducted by Pol δ. An alternative model developed in a recent study proposed that Pol δ is the major replicase for both strands (Johnson, Klassen, Prakash, & Prakash, 2015). It has also been reported that eukaryote GINS associates with DNA polymerase and stimulates the DNA polymerase activity (Chang et al., 2007). Dpb2, the non‐catalytic subunit of DNA polymerase epsilon (Pol ε), facilitates the interaction between Pol ε and GINS by associating with Psf1 and Psf3 (Garbacz et al., 2015; Grabowska et al., 2014). Furthermore, a gene mutation study of *Saccharomyces cerevisiae* cells proposed that GINS is related to replication fidelity via its facilitation of error‐free processing of the terminal mismatch created by Pol ε, while avoiding spontaneous mutagenesis and frameshifts formation during DNA replication (Grabowska et al., 2014).

Similar to eukaryotes, archaeal PolB is likely involved in leading strand synthesis, whereas PolD plays a key role in lagging strand synthesis in Thermococcales (Li, Kelman, & Kelman, 2013). Different from PolD binds to GINS15 (Figure 2), in this research, PolB does not bind to neither GINS15 nor GINS23 (Appendix Figure A1, panels K–M). Nevertheless, the attempt to delate the PolD and PolB genes from the genome of *T. kodakarensis* and *Methanococcus maripaludis* demonstrated that only PolD is essential for viability and that it may be the only replicative DNA polymerase required to replicate both the leading and lagging strand (Cubonova et al., 2013; Sarmiento, Mrazek, & Whitman, 2013). Although most DNA polymerase families have been well elaborated, studies of complexes containing PolD, which exist in all of the archaeal lineages with the exception of the Crenarchaeota (Cann et al., 1998), have been limited (Jokela et al., 2005). PolD is a heterodimer consisting of 3′–5′ exonuclease activity (DP1) and 5′–3′ polymerase activity (DP2) (Cann et al., 1998; Ishino et al., 1998; Uemori et al., 1997). The activity of each subunit requires the presence of another subunit, which is unique to the PolD family (Shen et al., 2004, 2003). Previous studies have shown that PolD exists in complexes with several replisome components, including minichromosome maintenance (MCM) helicase, DNA ligase, the archaeal Cdc45 protein, and proliferating cell nuclear antigen (PCNA) processivity factor (Kuba et al., 2012; Li et al., 2011, 2010; Motz et al., 2002). These data support that PolD is the key protein responsible for DNA replication.

**Figure 7:**
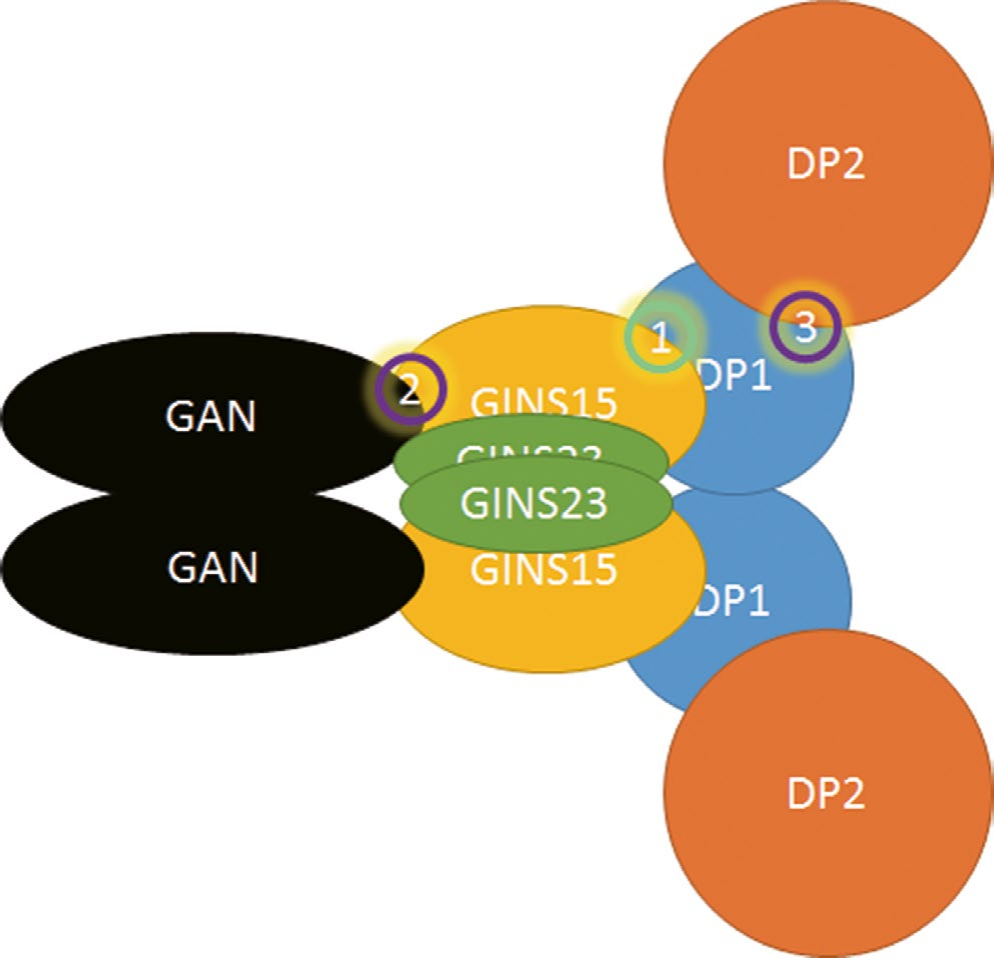
Proposed model for the architecture of the replisome in Euryarchaeota. The GINS complex of archaea is organized as GINS15_2_–GINS23_2 _(Oyama et al., 2011). The interaction between DP1 and DP2 subunits of PolD was previously reported [circle 3, (Uemori et al., 1997)]. The association between GAN and GINS15 was observed in our previous study ([circle 2], [Li et al., 2011]). The physical and functional interaction between GINS15 and DP1 (circle 1) was proposed (Pluchon et al., 2013) and further proved in the present study

In *Thermococcus* sp. 4557, we confirmed that DP1 and DP2 form a complex (Appendix Figure A1), which was in agreement with the results of a previous study. Interestingly, we found that DP1 directly interacts with GINS15, and that the latter inhibits the DNA 3′–5′ exonuclease activity of DP1 (Figures 4 and 6). This inhibited DP1 exonuclease, which is associated with the proofreading activity of PolD, strongly indicates that DP1 not only binds to, but also has the potential to be regulated by GINS15 in vivo. In PolD, the polymerase and proof reading exonuclease activities are possessed by DP2 and DP1 subunits individually. Compared with other DNA polymerases with polymerase and exonuclease activity centers in the same subunit, the PolD has higher possibility to have a separated DP1. The regulation of exonuclease activity is necessary to prevent its deleterious effects to cells. The observations from this study imply that in PolD, the exonuclease activity might be regulated by GINS15 in addition to DP2, the polymerase subunit. The mechanism of this GINS15–DP1 interaction is not yet known, therefore, further study is necessary. Moreover, the Biacore data also revealed that DP1 interacts with the GINS complex through GINS15, but not GINS23 (Figure 5). In our previous study, GAN, a 5′–3′ exonuclease, was found to bind to the GINS complex through the GINS15 subunit (Li et al., 2011). We propose that GINS15 serves as a bridge that connects GAN and DP1 to form a large complex. Another study also confirmed that the GINS tetramer contains two copies of the GINS15 and GINS23 subunits (Oyama et al., 2011). Based on this information, we propose a novel complex, PGG (including **P**olD, **G**INS, and **G**AN), which contains at least two GINSs, two GANs, two DP1s, and two DP2s (two PolDs). In this model, each protein component exists in a dimer (except DP2), as shown in Figure 7. DP1 binds directly to GINS15 to form GINS–PolDs complex (Figure 7), while the GAN dimer interacts directly with the GINS15 dimer (Figure 7) to make up a GAN–GINS complex. Hence, GINS serves as a bridge forming a large complex. Along with the observation that without PolB, the PolD is sufficient for DNA replication in *T. kodakarensis*, it is possible that *Thermococcus* sp. 4557 employs at least two PolDs on the DNA replisome complex. Since the *E. coli* replication fork was reported to have three Pol III cores (Kurth & O'Donnell, 2013), this will made it interesting to investigate the number of PolD cores in *Thermococcus* sp. 4557.

Many studies have indicated that archaeal GAN may act as a functional homolog of Cdc45 from eukaryotes, except that GAN has extra 5′–3′ exonuclease activity (Oyama et al., 2016). As a result, the PGG complex not only has DNA polymerase activity, but also possesses 5′–3′ exonuclease activity, resembling the bacterial DNA polymerase I containing 5′–3′ exonuclease activity, which is responsible for the removal of RNA primers from Okazaki fragments. In eukaryotes and archaea, it is believed that Fen1 is involved in removing the RNA primers from the Okazaki fragments (Bambara, Murante, & Henricksen, 1997; Henneke, 2012). In most of Euryarchaea including *T. kodakarensis*, genes encoding both Fen1 and GAN were identified from the genome. Although individual genes can be removed from the genome, the double mutation of GAN plus Fen1 and GAN plus RNAse HII were not successful (Burkhart et al., 2017). This observation implies that GAN is in a parallel pathway of the known Fen1‐RNAse HII in archaea. In view of these data, we speculate that GAN in the PGG complex is probably sufficient for primer processing during Okazaki fragment maturation. However, it is also important to note that, when compared with bacterial RecJ, tkGAN has a limited 5′–3′ exonuclease activity in the presence of magnesium (Li et al., 2011). This might imply that GAN was losing its role in Okazaki fragment maturation and finally became a scaffold protein functioning as eukaryotic Cdc45, which has no exonuclease activity. Fen1 with RNAse HII as a successor plays the role of GAN in the Okazaki fragment processing in the more complex eukaryote cells.

## 5 CONCLUSION

In conclusion, we identified a complex designated PGG that contains DNA polymerase PolD, GINS, and GAN. We assume that this complex might be the DNA replication core, in which GAN is responsible for the Okazaki fragment processing, and that this role was finally succeeded by Fen1. Two copies of PolD are working on both leading strand and lagging strand synthesis, while GINS are the scaffold that holds GAN and PolD together. Further studies will be conducted to address the other components that might interact with the PGG complex, including the replicative helicase MCM and the ring‐shaped PCNA.

## CONFLICT OF INTERESTS

None declared.

## AUTHOR CONTRIBUTIONS

Shuhong Lu, Zhuo Li, Yulong Shen, Xuesong Zhang, Kaiying Chen, Zimeng Chen, Yixiang Li, and Zhongquan Qi conceptualized the study. Shuhong Lu and Zhuo Li managed the data curation. Shuhong Lu and Zhuo Li formally analyzed the data. Zhuo Li acquired the funding. Shuhong Lu, Zhuo Li, Yulong Shen, Xuesong Zhang, Kaiying Chen, Zimeng Chen, Yixiang Li, and Zhongquan Qi investigated the study. Shuhong Lu, Zhuo Li, Yulong Shen, Xuesong Zhang, Kaiying Chen, Zimeng Chen, Yixiang Li, Zhongquan Qi designed the methodology. Zhuo Li managed the project. Shuhong Lu and Zhuo Li provided the resources for the study. Zhuo Li supervised the study. Shuhong Lu and Zhuo Li validated the study data. Shuhong Lu and Zhuo Li visualized the study. Shuhong Lu, Zhuo Li, and Yulong Shen wrote the original draft of the manuscript. Shuhong Lu, Zhuo Li, Yulong Shen, Xuesong Zhang, Kaiying Chen, Zimeng Chen, Yixiang Li, and Zhongquan Qi reviewed and edited the manuscript.

## ETHICS STATEMENT

None required.

## DATA ACCESSIBILITY

All data supporting this study is provided as appendices information accompanying this paper.

## Figures and Tables

**Figure A2:**
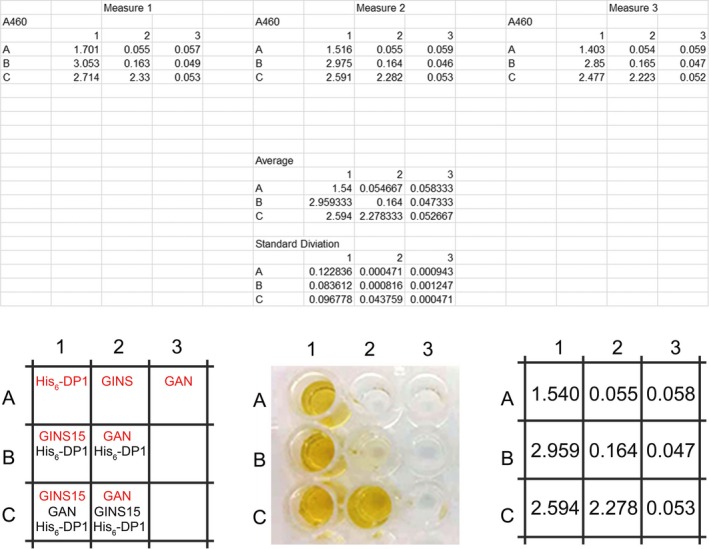
Measurements of HRP signal strength and calculations of pull‐down assay

## APPENDIX 1

**Table A1:** Oligonucleotides used for gene cloning from *Thermococcus *sp. 4557

Proteins	GenBank ID	Primers Names	Sequences^a^
GINS15	AEK72061.1	GINS15‐F	GATATA*CATATG*GACATCGTCAAGCTCAGGGAAC
		GINS15‐R	GCTGTAGTCGACTCACGGGGCGATCCTTATCCTCT CG
GINS23	AEK72908.1	GINS23‐F	GATATACATATGTTCACGGGGAGGGCAGTGATAC
		GINS23‐R	GCTGTAGTCGACTCACTCCTCACCGAGCCATTCGT
GAN	AEK72413.1	GAN‐F	GATATACATATGGATAGAGAGGCGTTTTTGGAGCG
		GAN‐R	GCTGTAGTCGACTCAATCCTCACCGGATTTTC
PolD‐DP1	AEK73338.1	PolD‐DP1‐F	GATATACATATGGGTTTGATCGAGGATTTAATGGC
		PolD‐DP1‐R	GCTGTAGTCGACTCAAACCCCCTCACAGTACTCGT CAAAC
PolD‐DP2	AEK73341.1	PolD‐DP2‐F	GATATACATATGCCTGAGCTCTACTCATCCGAGATG AAATC
		PolD‐DP2‐R	GCTGTAGTCGACCTAAGAGCCGAAGAACTCGTCG AGGCTTATG
		PolD‐DP2‐M1^b^	GTCTTCATCTCCGTCCCTCCTCTTCGCTGCGTGAT AATACGGGTGCGCGTAG
		PolD‐DP2‐M2^b^	ACGCAGCGAAGAGGAGGGACGGAGATGAAGACG CTGTAATGCTCCTCCTCG

a Underlines indicates the restriction sites used in cloning the fragments in to modified pET15b.

b Primers used for removing intein coding sequences within PolD‐DP2.
